# Targeted exosome-mediated delivery of opioid receptor Mu siRNA for the treatment of morphine relapse

**DOI:** 10.1038/srep17543

**Published:** 2015-12-03

**Authors:** Yuchen Liu, Dameng Li, Zhengya Liu, Yu Zhou, Danping Chu, Xihan Li, Xiaohong Jiang, Dongxia Hou, Xi Chen, Yuda Chen, Zhanzhao Yang, Ling Jin, Waner Jiang, Chenfei Tian, Geyu Zhou, Ke Zen, Junfeng Zhang, Yujing Zhang, Jing Li, Chen-Yu Zhang

**Affiliations:** 1 State Key Laboratory of Pharmaceutical Biotechnology, NJU Advanced Institute of Life Sciences, Jiangsu Engineering Research Center for MicroRNA Biology and Biotechnology, School of Life Sciences, Nanjing University, Nanjing 210093, China

## Abstract

Cell-derived exosomes have been demonstrated to be efficient carriers of small RNAs to neighbouring or distant cells, highlighting the preponderance of exosomes as carriers for gene therapy over other artificial delivery tools. In the present study, we employed modified exosomes expressing the neuron-specific rabies viral glycoprotein (RVG) peptide on the membrane surface to deliver opioid receptor mu (MOR) siRNA into the brain to treat morphine addiction. We found that MOR siRNA could be efficiently packaged into RVG exosomes and was associated with argonaute 2 (AGO2) in exosomes. These exosomes efficiently and specifically delivered MOR siRNA into Neuro2A cells and the mouse brain. Functionally, siRNA-loaded RVG exosomes significantly reduced MOR mRNA and protein levels. Surprisingly, MOR siRNA delivered by the RVG exosomes strongly inhibited morphine relapse via the down-regulation of MOR expression levels. In conclusion, our results demonstrate that targeted RVG exosomes can efficiently transfer siRNA to the central nervous system and mediate the treatment of morphine relapse by down-regulating MOR expression levels. Our study provides a brand new strategy to treat drug relapse and diseases of the central nervous system.

RNA interference (RNAi) refers to guide sequence-dependent gene silencing mediated by either the degradation or translation arrest of target RNAs^1^. The discovery of small-interfering RNA (siRNA) as a mediator of RNAi in mammalian cells rapidly brought RNAi to the forefront as a promising tool for therapeutic applications in cancers and other diseases^2^,^3^. The delivery of siRNA remains a challenging task, and tissue-specific delivery of siRNA will bring RNAi therapy more clinic al value. Thus, finding an effective siRNA delivery tool for therapeutic administration *in vivo* is a problem that urgently needs to be addressed. Three types of delivery vehicle have been used for siRNA delivery, including viruses, polycationic polyethylenimine (PEI)-based nanoparticles and liposomes^4^,^5^,^6^,^7^. Nonetheless, there are still some disadvantages with each method, including immune activation, toxicity problems and non-specific targeting^1^,^2^,^3^. Thus, an efficient, tissue-specific and non-immunogenic delivery tool must be developed.

Microvesicles (MVs), with diameters ranging from 30 to 1000 nm, are secreted from almost all cell types under both physiological and pathological conditions^4^,^5^. MVs can be divided into two types: exosomes and shedding vesicles^5^. MVs released from cells have been shown to contain non-coding RNAs, which can be transferred to neighbouring or distant cells to regulate the gene expression of recipient cells^6^. Our previous study demonstrated that MVs could be utilised as a delivery vehicle to transport therapeutic siRNA or anti-sense microRNA for tumour therapy, indicating the potential of MVs as a tool for tumour treatment^7^,^8^,^9^. However, the utilisation of MV-delivered siRNA for the treatment of other diseases has not been explored. MVs can also be engineered to express specific ligands on the membrane surface; these artificially modified MVs can then enter into specific tissues. Lydia *et al*. acquired targeted exosomes by engineering the exosomes from dendritic cells to express the neuron-specific rabies viral glycoprotein (RVG) peptide, which binds to the acetylcholine receptor expressed on neuronal cells, to allow these exosomes to efficiently pass through the blood-brain barrier (BBB)^10^. Thus, the RVG-modified exosomes allow for the delivery siRNA into the brain.

In the current study, we utilised RVG exosomes loaded with opioid receptor Mu (MOR) siRNA to treat drug addiction via down-regulating the expression of MOR, which is the primary target for opioid analgesics used clinically, including morphine, fentanyl and methadone, and is involved in the primary reinforcing effects of and the addiction to opiates. Here, we selected the human embryonic kidney 293T (HEK 293T) cell line and co-transfected the cells with an RVG peptide-expressing plasmid and MOR siRNA to acquire RVG exosomes loaded with MOR siRNA. Moreover, we analysed MOR expression levels *in vitro* and *in vivo* and morphine relapse in mice. Our study provides a brand new strategy for treating drug addiction.

## Results

### Characterisation of RVG exosomes and the packaging of MOR siRNA into RVG exosomes

**Figure 1:**
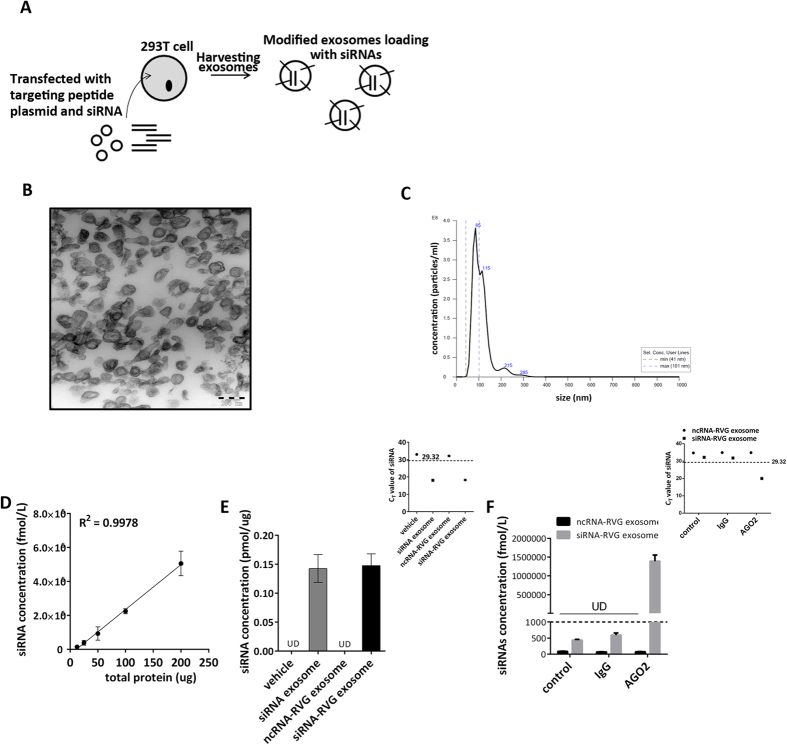
The characterisation of RVG exosomes loaded with MOR siRNA. (**A**) Schematic diagram of the production and harvest of RVG-modified exosomes for siRNA delivery. (**B**) TEM micrographs of RVG exosomes isolated from the culture medium of 293T cells. (**C**) Exosomes were measured by using Nanosight NS 300 system in the supernatant from cultures cells. The histogram represents particle size distribution. (**D**) qRT-PCR analysis of siRNA levels in various quantities of exosomes. *P < 0.05; **P < 0.01. (**E**) qRT-PCR analysis of MOR siRNA levels in various exosomes. (**F**) qRT-PCR analysis of MOR siRNA levels in anti-AGO2 or anti anti-IgG immunoprecipitated products from RVG exosomes treated with or without AGO2 antibody. RVG-modified exosomes with or without siRNA were isolated from 293T culture medium and immunoprecipitated with or without anti-AGO2 antibody. Then, MOR siRNA levels in immunoprecipitated products from RVG exosomes were assayed by qRT-PCR.

The effects of many neuropharmaceuticals are diminished by the presence of the BBB. So far, there is no solid evidence that exosomes can pass through the BBB to enter the brain. To acquire modified exosomes that can pass through the BBB, we established neuron-specific exosomes according to a previous publication^10^. First, the RVG peptide was cloned into Lamp2b, a protein expressed abundantly in exosomal membranes. Then, the plasmids encoding RVG and MOR siRNA were simultaneously transfected into HEK 293T cells for 48 hr before exosomes were collected (Fig. 1A). Isolated exosomes were characterized using transmission electron microscopy (TEM) and NTA. The TEM photographs showed that the exosomes presented normal morphological characteristics, with a diameter of approximately 90 nm, and that each vesicle was surrounded by a double-layer membrane; the NTA results showed that the diameter of majority of particles are 85 nm. These characteristics indicate that the exosome properties were not affected by the modifications (Fig. 1B,C). To identify the interference efficiency of the MOR siRNA, the mouse neuroblastoma cell line (Neuro2A) was transfected with MOR siRNA via liposomes, resulting in a great reduction of MOR mRNA (supplementary Fig. 1A). The three MOR siRNAs have the same interference, thus, we select siRNA-1 and siRNA-2 for the next experiment. Next, the levels of MOR siRNA in isolated exosomes were assayed by a LNA primer-based quantitative RT-PCR assay. The serially diluted MOR siRNAs were assessed using the qRT-PCR assay to generate a standard curve. The siRNA had a Pearson correlation coefficient (R) >0.99 (supplementary Fig. 1B). The linear range of the C_T_ value was from 16.86 to 29.32, and the corresponding quantification range of the expression level was from 10 amol to 100 fmol (supplementary Fig. 1B and Table 1). The siRNAs concentration in exosomes was calculated based on reference to the standard curve and is linearly and positively correlated with the total number (shown as total protein) of exosomes (Fig. 1D). In vehicle and RVG-exosome loaded with scramble RNA (ncRNAs), the C_T_ values of siRNAs (quantified using MOR siRNAs probe) were outside the linear range, thus, this C_T_ values were the background values of qRT-PCR assay and the siRNAs in these exosome were undetected. The final concentration of siRNA in exosome loaded with them were approximately 0.14 pmol/μg (Fig. 1E). The results clearly showed that MOR siRNA can be effectively packaged into exosomes.

### MOR siRNA is associated with AGO2 in RVG exosomes

Previous publications showed that miRNA and siRNA in exosomes were combined with the argonaute 2 (AGO2) complex^7^. Thus, we next determined whether MOR siRNA was associated with AGO2 in RVG-exosomes. The association of MOR siRNA with AGO2 was detected by AGO2 immunoprecipitation using an anti-AGO2 or anti-IgG antibody, followed by analysis of the MOR siRNAs. The result showed that the majority of the MOR siRNA was associated with AGO2 (Fig. 1F). Taken together, these results demonstrate that MOR siRNA can be effectively packaged into RVG exosomes and is associated with AGO2 in RVG exosomes.

### RVG exosomes can specifically deliver siRNA into Neuro2A cells and reduce MOR expression levels in the recipient cells

**Figure 2:**
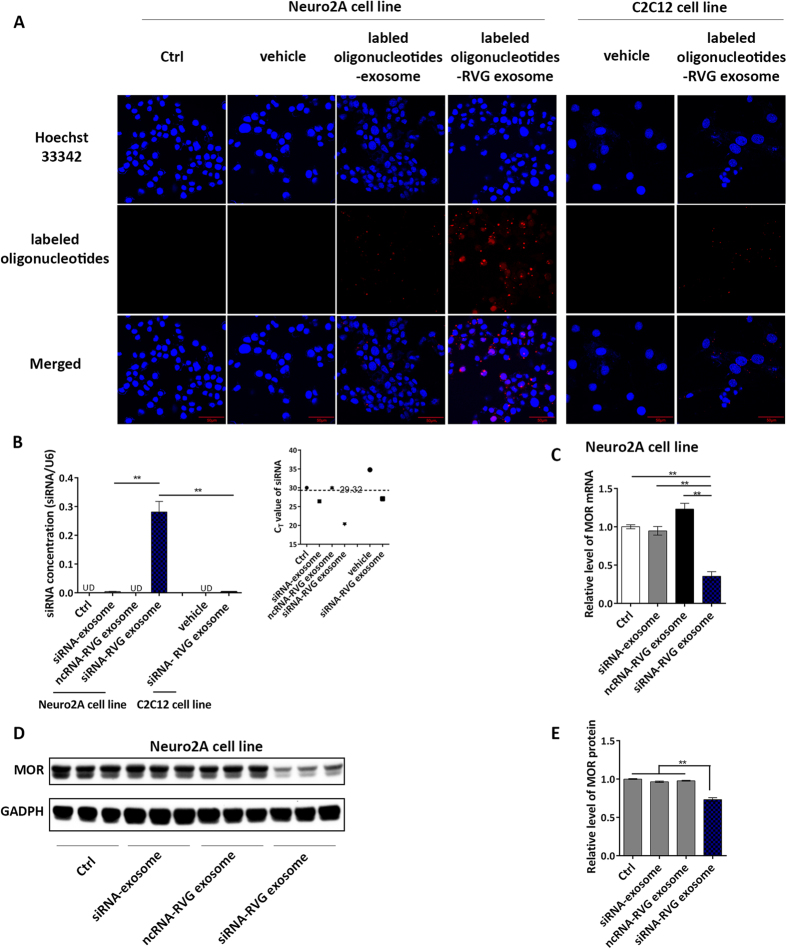
RVG exosome-delivered siRNA can enter Neuro2A cells and reduce MOR expression levels. (**A**) Confocal microscopy images of fluorescently labelled oligonucleotides in Neuro2A cells and C2C12 cells, untreated (lane 1 and 5) or incubated with empty exosome (lane 2), normal exosomes loaded with siRNA (lane 3) or RVG exosomes loaded with siRNA (lane 4 and 6). (**B**) qRT-PCR analysis of MOR siRNA concentration in Neuro2A cells and C2C12 cells untreated or treated with siRNAs in normal exosome, scramble RNAs in RVG exosome or siRNA in RVG exosome. (**C**) qRT-PCR analysis of MOR mRNA levels in Neuro2A cells treated as described in (**B**). (**D**) Western blot analysis of MOR protein levels of Neuro2A cells treated as described in (**B**). (**E**) Quantification of the MOR protein levels in (**A**). *P < 0.05; **P < 0.01.

To determine whether RVG exosomes can deliver small RNAs into cells, the Neuro2A cell line was selected as the recipient cell to incubate with exosomes loaded with or without Alexa Fluor 555 labelled oligonucleotide (show red colour). As shown in Fig. 2A, Neuro2A cells treated with RVG exosomes loaded with Alexa Fluor 555-labelled oligonucleotide (lane 4) were fluorescently labelled under fluorescence confocal microscopy. The fluorescent signals were not observed in cells untreated or treated with vehicle or non-RVG exosome (lane 1, 2 and 3). Interestingly, we found that RVG exosomes only entered neurocytes, which have the RVG peptide receptor on their membrane; these exosomes could not efficiently enter other non-neuronal cells such as the human skeletal muscle cell line (C2C12) (Fig. 2A lane 5 and 6). Subsequently, MOR siRNA levels were assayed in recipient cells. siRNAs were detected in Neuro2A cells after treatment with RVG exosomes loaded with MOR siRNA (siRNA-RVG exosomes). The siRNAs concentrations were barely detected in Neuro2A treated with siRNAs in normal exosome. Similarly, the siRNAs barely detected in C2C12 cells treated with RVG-exosome (Fig. 2B). Consistently, MOR mRNA and protein levels were dramatically reduced by RVG exosome-delivered siRNA (siRNA-RVG exosomes), while no reduction in the MOR mRNA and protein levels were observed with the exosomes without the RVG peptide (siRNA exosomes) (Fig. 2C–E). Scramble RNAs (ncRNAs) delivered by RVG-exosome also could not reduce MOR mRNA and protein levels, suggesting that the reduction of target gene was mediated by siRNAs (Fig. 2C–E). To confirm the effect of MOR siRNAs loaded RVG-exosome *in vitro*, a second siRNA (siRNA-2) was used. As shown in Supplementary Figure 2A and 2B, siRNA-2 encapsulated in RVG-exosome also could reduce MOR expression level. Together, these results clearly demonstrate that the RVG peptide modification on the exosome membrane specifically guides exosomes to target cells bearing the RVG peptide receptor, allowing for the efficient delivery of MOR siRNA into the recipient cells to regulate MOR gene expression.

### RVG exosomes can transfer siRNAs through the BBB and reduce MOR expression levels

**Figure 3:**
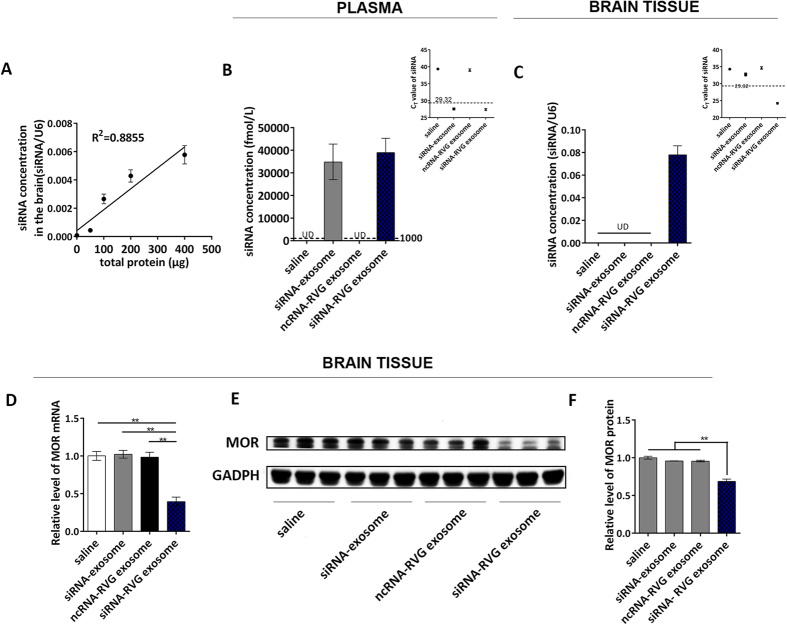
siRNA-RVG exosomes reduced MOR levels in mice. (**A**) qRT-PCR analysis of MOR siRNA levels in the brains of mice following injection with various quantities of exosome. (**B**) qRT-PCR analysis of MOR siRNA concentration in the plasma of mice untreated or treated with empty exosomes, siRNAs in normal exosome, scramble RNAs in RVG exosome or siRNAs in RVG exosome. (**C**) qRT-PCR analysis of MOR siRNA concentration in the brains of mice following intravenous injection as described in (**B**). (**D**) qRT-PCR analysis of MOR mRNA levels in the brains of mice treatment as described in (**B**). (**E**) Western blot analysis of MOR protein levels in the brains of mice treated as described in (**B**). (**F**) Quantification of the MOR protein levels in (**B**). n = 7, *P < 0.05; **P < 0.01.

To verify whether siRNA delivered via RVG exosomes can pass through the BBB and regulate MOR expression, we performed an *in vivo* assay. Mice were injected once intravenously with siRNA-RVG exosomes, and the siRNA levels in the brain were assayed after 24 hours. The result showed that 200 μg exosomes per mouse produced the greatest elevation in siRNA levels in the brain; thus, a 200 μg dose was chosen for the following experiments (Fig. 3A). siRNAs were detected in the mice plasma injected with siRNAs loaded in normal exosomes or RVG exosomes (Fig. 3B) and the final siRNA concentration in plasma was determined as 3.89 fmol/μl (supplementary table 1). Interestingly, only RVG exosomes could delivery siRNAs into brain and significantly reduce MOR mRNA and protein levels (Fig. 3C–F). And the final concentration of siRNAs in brain tissue was determined as 18.82 pmol/g (supplementary table 1). To assay the MOR siRNAs distribution in the brain, we sub-dissect the brain into cortex, hippocampus, thalamus, hypothalamus, striatum. MOR siRNAs were detectable in all these regions and have a highest level in the cortex, where MOR are majorly expressed and regulates the opioid addiction. (Supplementary Fig. 3A). Scramble RNAs (ncRNAs) delivered by RVG exosome could not reduce MOR mRNA and protein levels, suggesting that the reduction of target gene was mediated by siRNAs (Fig. 3D–F). To further confirm the effect of MOR siRNAs loaded in RVG-exosome *in vivo*, a second siRNA (siRNA-2) was used. As shown in Supplementary Figure 3B and 3C, injection of siRNA-2 encapsulated in RVG-exosome could also reduce MOR expression level in the brain. Taken together, these results clearly demonstrate that exosomes with RVG modification passed through the BBB and delivered siRNA into the central nervous system to regulate gene expression, while natural exosomes without the RVG modification were not capable of delivering siRNA into the central nervous system or regulating target gene expression.

### The effects of siRNA delivered by RVG exosomes on morphine-induced CPP

**Figure 4:**
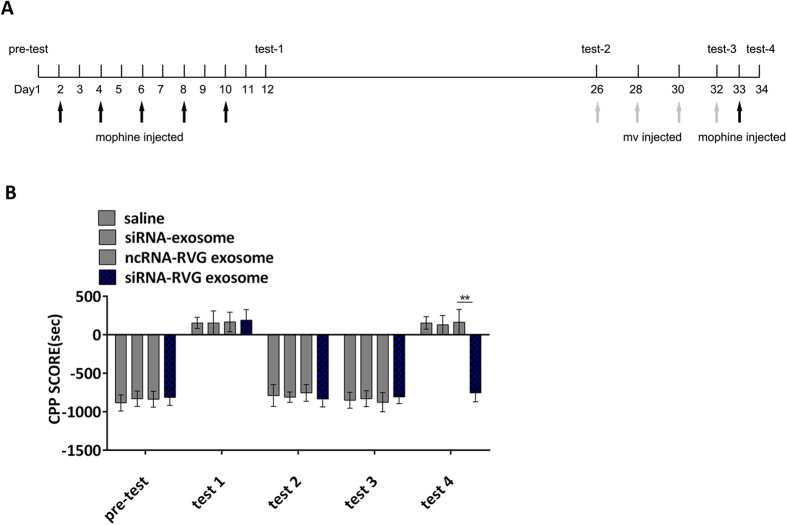
The effects of siRNA-RVG exosomes on morphine-induced CPP. (**A**) A flow chart depicting the experimental design is shown. (**B**) Analysis of the morphine-reduced CPP of mice treated with saline, empty exosomes, scramble RNAs in RVG exosomes or siRNAs RVG exosomes. n = 7, *P < 0.05; **P < 0.01.

We then investigated the consequences of MOR down-regulation in the animals by conducting the conditioned position preference (CPP) test. MOR and its signalling pathway are known to be involved in drug relapse of opiates such as morphine and heroin. Importantly, relapse always disrupts the process of drug withdrawal. Thus, our current study focused on the influence of MOR siRNA on drug relapse. In the CPP paradigm, mice learned to associate the rewarding effect of morphine with a drug-paired environment. The CPP test was performed as depicted in Fig. 4A. Before conditioning, the mice showed a preference for visiting black chamber. Then, morphine dependence was developed by i.p. injection of morphine, given every other day for a total of 5 times. Mice were place-conditioned by i.p. injection with morphine in the white chamber on even-numbered days (on days 2, 4, 6, 8, 10) and with saline in the black chamber on odd-numbered days (on days 3, 5, 7, 9, 11). Each injection was performed 5 times. On day 12, CPP test 1 was conducted by allowing the mice to freely visit the morphine- and saline-paired chambers; mice showed a significant preference in visiting the morphine-paired chamber, suggesting the development of morphine dependence. Then, morphine and saline treatments were removed for several days. On day 26, CPP test 2 was conducted and mice spent less time in the morphine-paired chamber than the saline-paired chamber, suggesting the disappearance of morphine dependence. Then, mice were treated with the various exosomes intravenously once every two days for a total of 4 times. Then, CPP test 3 was performed on day 32. Mice still showed a natural preference for the black chamber, suggesting that MOR siRNA had no effect on the behaviour of the mice. Then, mice were relapsed on morphine on day 33; CPP test 4 was performed the next day. Interestingly, the mice treated with RVG exosome-delivered siRNAs (siRNA-RVG exosomes) still showed a natural preference for the black chamber, while the mice treated with saline, siRNAs loaded in normal exosome or ncRNA in RVG exosome show preference to drug-paired chamber, suggesting that the MOR siRNAs delivered by RVG exosome restrain drug addiction when the mice were re-exposed to morphine (Fig. 4B). A second siRNA (siRNA-2) was used to further confirm the effect of MOR siRNAs loaded in RVG-exosome on morphine-induced CPP. The results showed that siRNA-2 packaged in RVG-exosome could also restrain the morphine relapse (Supplementary Fig. 4).

## Discussion

Exosome research has recently gained attention. Our previous study demonstrated that exosomes are functional vesicles that contain post-transcriptional RNA (miRNA) and mediate cell-to-cell communication via their miRNA content^6^,^11^. The following study further demonstrated that exogenous siRNA transfected into cells could also be packaged by exosomes and delivered into recipient cells to regulates gene silencing, indicating that exosomes can serve as siRNA delivery vesicles in gene therapy for cancer and other diseases^12^,^13^,^14^. The development of tissue-specific delivery vesicles represents great progress in gene therapy. Thus, upon learning of exosomes expressing the neuron-targeting RVG peptide on their surface, we wanted to utilise this targeted exosome to solve medical problems in the brain. Drug addiction poses serious social, medical, and economic issues, but effective treatments for drug addiction remain limited^15^,^16^. Thus, we proposed the bold strategy of using RVG exosomes to deliver siRNA into the brain to treat drug addiction. MOR is a major target of opioid drugs and appears to play critical roles in mediating the major effects of opioid drugs, including analgesia, tolerance, abuse, dependence and respiratory depression^17^. It has been reported that the rewarding effect of morphine, mediated by MOR, is abolished in MOR-deficient mice and that an MOR antagonist diminished the consequences of an initial opioid drug relapse^18^. Thus, the MOR gene was selected as a target for the treatment of drug addiction. Here, according to method described in our previous publication^10^, we constructed a plasmid expressing the neuron-targeting peptide RVG and employed HEK 293T cells as donor cells to acquire exosomes expressing RVG on the surface. One of two methods, electroporation or transfection, is commonly used to load exosomes/MVs with exogenous siRNA. The process of donor cell transfection for loading therapeutic cargo into exosomes is generally more effective than direct electroporation. In the current study, we demonstrated that the second method has a very high efficiency for siRNA loading; approximately 1% of the siRNA in the donor cells could be packaged into exosomes, which is a much higher than that of electroporation. The exosomes showed normal morphological characteristics after modification and siRNA loading were conducted. Argonaute proteins are the central protein component of RNA-induced silencing complexes (RISCs); it has been reported that only the siRNAs associated with AGO2 are functional and stable^19^. In the current study, we demonstrated that siRNAs were combined with AGO2 in RVG-modified exosomes, indicating that these siRNAs were functional and stable.

According to the previous study, the RVG peptide could mediate exosome transfer through the BBB via binding to the acetylcholine receptor. Our study confirmed that RVG exosomes bearing MOR siRNA could effectively enter cells expressing the acetylcholine receptor on their membranes, resulting in a great reduction in the mRNA and protein levels of the target gene *in vitro*. Interestingly, RVG exosomes could not effectively enter cells lacking the acetylcholine receptor and did not reduce the MOR mRNA and protein levels in these cells, further demonstrating that RVG exosomes represent a class of vesicles that can specifically and effectively enter neurons that express the acetylcholine receptor. The animal experiments demonstrated that RVG exosomes bearing MOR siRNA could pass through the BBB, enter the brain and significantly decrease MOR gene expression levels. In addition, the CPP test was conducted to determine the effect of siRNA delivered via RVG exosomes on drug relapse. Surprisingly, the results showed that MOR down-regulation by RVG exosome-delivered siRNA clearly blocked the morphine-induced CPP after re-exposure to morphine, indicating that the siRNA prevented morphine relapse. On the contrary, RVG exosomes loaded with siRNA had no effect on the natural preference of the mice not treated with morphine. Notably, siRNA may serve as a more effective treatment for drug addiction compared with other options such as naltrexone and methadone.

Great progress has been made in finding the perfect delivery vesicles for gene therapy. Exosomes are thought to have unequivocal advantages compared with traditional delivery systems such as liposomes, viruses and nanoparticles because of their non-toxicity and non-immunogenicity. In this study, we utilised brain-specific targeted exosomes to solve a pathological problem in the central nervous system (CNS) and demonstrated that exosomes have the potential to serve clinically as a gene therapy strategy for the transfer of siRNA across the blood brain barrier.

## Materials and Methods

### Cell lines and reagents

The human embryonic kidney 293T, mouse skeletal muscle C2C12 and mouse Neuro2A cell lines were purchased from the Institute of Biochemistry and Cell Biology, Shanghai Institutes for Biological Science, Chinese Academy of Sciences (Shanghai, China). Both cell lines were cultured in high-glucose DMEM medium, supplemented with 10% FBS (Gibco, CA, USA) and penicillin (100 U/ml)/streptomycin (100 mg/ml), at 37 °C in 5% CO2. Anti-MOR, anti-GAPDH and anti-IgG antibodies were purchased from Santa Cruz Biotechnology (Santa Cruz, CA, USA), and an anti-Ago2 antibody was purchased from Abcam (Cambridge, MA, USA).

### Transfection and preparation of exosomes

The MOR siRNA oligonucleotides were synthesised by Invitrogen. The sequences of the siRNAs duplex are siRNA-1: sense 5′-3′ ACUUCCUCCACAAUCGAACAGCAAA; antisense, antisense 5′-3′ UUUGCUGUUCGAUUGUGGAGGAAGU; siRNA-2: sense 5′-3′ GUCAUGUAUGUGAUUGUAAGAUA, antisense 5′-3′ UAUCUUACAAUCACAUACAUGAC; siRNA-3: sense 5′-3′ GCAAGAUCGUGAUCUCAAUAGACUA, antisense 5′-3′ UAGUCUAUUGAGAUCACGAUCUUGC. As previously described^10^, the targeting peptide RVG was cloned into Lamp2b, a protein that is abundantly expressed on the exosome membrane surface. 293T cells were seeded in 225-cm^2^ flasks (Corning). When the cells reached approximately 70–80% confluence, they were cotransfected with siRNA and the RVG-Lamp2b plasmid using Lipofectamine 2000 (Invitrogen) according to the manufacturer’s instructions. The cell culture medium was then harvested 48 h after transfection, and exosomes were isolated from the medium using an exosome isolation kit (Invitrogen) according to the manufacturer’s instructions. The resulting pellet was then resuspended in saline. Alexa Fluor 555 fluorescence-labelled siRNA oligonucleotides was purchased from Invitrogen. 293T cells were transfected with the labelled siRNA and exosomes loaded with fluorescence-labelled siRNA were harvested as above described. Neuro2A and C2C12 cells were transfected MOR plasmid using lipofectamine 2000 to expression MOR as above described.

### Transmission electron microscopy assay

For the TEM assay, the exosome samples were prepared as above described. Briefly, the exosome pellet was placed in a droplet of 2.5% glutaraldehyde in PBS buffer and fixed overnight at 4 °C. The exosome samples were rinsed 3 times in PBS for 10 min each and then fixed in 1% osmium tetroxide for 60 min at room temperature. Then, the samples were embedded in 10% gelatine, fixed in glutaraldehyde at 4 °C and cut into small blocks (less than 1 mm^3^). The samples were dehydrated in increasing concentrations of alcohol. Then, the samples were placed in propylene oxide and infiltrated with increasing concentrations of Quetol-812 epoxy resin mixed with propylene oxide for 3 h per step. Finally, the samples were embedded in pure, fresh Quetol-812 epoxy resin and polymerised at 35 °C for 12 h, 45 °C for 12 h, and 60 °C for 24 h. Ultrathin sections were cut using a Leica UC6 ultra-microtome and stained with uranyl acetate for 10 min and lead citrate for 5 min at room temperature. The samples were then observed with a transmission electron microscope (JEM-1010) at a voltage of 80 kV.

### NTA

NTA was carried out using the Nanosight NS 300 system (NanoSight) on exosomes resuspended in saline at a concentration of 5 μg of protein/ml and were further diluted from 100- to 500-hundredfold for analysis. The system focuses a laser beam through a suspension of the particle of interest. The results are visualized by light scattering.

### Confocal microscopy analysis

Exosomes (100 μg exosome for 10^6^ cells) loaded with or without fluorescence-labelled siRNA were incubated with the Neuro2A cells. After 6 hours, the cells were washed, fixed and observed under a confocal microscope (FV 1000; Olympus, Tokyo). Pictures were taken under the following conditions: objective lens: PLAPON 60 × O; NA: 1.42; scan mode: XY; excitation wavelength: 405 nm for hoechst 33342 and 555 nm for Alexa Fluor 555; and image size: 1024 × 1024 pixels.

### Co-immunoprecipitation

Co-immunoprecipitation assays were performed according to the manufacturer’s instructions. Briefly, cells were washed three times with cold PBS (4 °C), scraped from each dish and then collected by centrifugation at 1000 rpm for 5 min at 4 °C). Cells were then re-suspended in an appropriate volume of complete lysis buffer. Mouse monoclonal anti-AGO2 antibody (2 μg) was used to immunoprecipitate RNA-binding proteins. After purification, immunoprecipitated RNA was analyzed by real-time RT-PCR for MOR siRNA using custom LNA siRNA primer (Exiqon, Denmark).

### Exosome incubation with cells

Exosomes (100 μg exosome for 10^6^ cells) loaded without or with MOR siRNAs, scramble RNAs were incubated with Neuro2A or C2C12 cells which were prior expressed MOR as described in 2.3. After 24 hour incubation, the recipient cells were collected for the following analysis.

### RNA isolation, quantitative RT-PCR and calculation

Total RNA from cell and tissue was extracted using TRIzol Reagent (Invitrogen) according to the manufacturer’s instructions. Total RNA from plasma was extracted using the miRneasy mini kit (Qiagen, Valencia, CA, USA). Quantitative RT-PCR of mature miRNA was performed on an ABI7500 (Applied Biosystems; Foster City, CA) instrument using the custom LNA siRNA primer (Exiqon) according to the manufacturer’s instructions. Briefly, 2 μl (plasma) or 10 ng (brain tissue) of total RNA was reverse-transcribed to cDNA using universal cDNA synthesis kit (Exiqon) and 4 μl cDNA (80 times diluted) was used for Real-time PCR using ExiLENT SYBR Green master mix kit. After the reaction, the cycle threshold (C_T_) values were determined using the threshold setting. To calculate the expression levels of the target siRNAs, a series of siRNA oligonucleotides at known concentrations in water were also reverse-transcribed and amplified to generate a stand curve. The quantification of siRNA was then calculated by referring to the standard curve^20^. The siRNA concentration in brain tissue was normalized to U6 RNA. Based on the calculations of the concentrations of siRNA in plasma, exosome and brain tissue, the final amount of siRNA were determined. Quantitative RT-PCR of MOR expression was also performed on an ABI7500 with primers purchased from Invitrogen. The primers used were as follows: MOR-1: 5′-GCCTTAGCCACTAGCACG-3′ (forward primer) and 5′-AACATTACGGGCAGACCA-3′ (reverse primer); and GAPDH: 5′-CGAAGGTGGAAGAGTGGGAG-3′ (forward primer) and 5′-TGAAGCAGGCATCTGAGGG-3′ (reverse primer).

### Western blots and antibodies

Tissues and cells were lysed in RIPA lysis buffer. MOR-1 protein levels were quantified by Western blot analysis using antibodies against MOR-1 (Santa Cruz). Normalisation was conducted by blotting the same samples with an antibody against GAPDH (Santa Cruz).

### Animals

C57BL/6J (male, 8-week-old) mice were purchased from the Model Animal Research Centre (MARC) of Nanjing University (Nanjing, China) and were maintained in pathogen-free conditions. The animals received humane care according to the guidelines prepared by the National Institutes of Health Guide for the Care and Use of Laboratory Animals and approved by the Institutional Review Board of Nanjing University, Nanjing, China. Mice were intravenously injected with 200 μg exosomes for once. After 6 hours, plasma siRNAs levels were assayed, and after 24 hours, siRNAs, MOR mRNA and protein levels were assayed in the brain tissue.

### Conditioned place preference (CPP) test

A two-chamber CPP apparatus (Yishu Software Technology Co. Ltd., Shanghai, China) was used in this study. The two chambers were identical in size but differed in colour and floor texture. The two distinct chambers were linked by a smaller intermediate compartment with a shutter on each side that allowed for control of access to either side of the chamber. One chamber had white walls with a barred floor and was illuminated, while the other had black walls with a dotted floor and was not illuminated.

#### *Pre-conditioning*

(Day 1) Mice were allowed free access to both chambers for 30 min, and the number of crossings and the time spent in each chamber were recorded. As recommended by the manufacturer’s instructions, animals with fewer than 20 crossings or less than 300 s spent in one chamber were excluded.

#### *Conditioning*

(Days 2–11) On days 2, 4, 6, 8 and 10, all mice received a morphine hydrochloride injection (10 mg/kg, i.p; Northeast Pharmaceutical group, Shenyang NO. 1 Pharmaceutical CO., LTD, China) before being placed in the white chamber for 30 min. On days 3, 5, 7, 9 and 11, all mice received a saline injection (10 ml/kg, i.p.) before being placed in the black chamber for 30 min. On day 12, all mice were allowed free access to both chambers for 30 min for the CPP test.

#### *Extinction*

(Days 13–25) All mice were kept in their cages and were maintained in pathogen-free conditions.

#### *Exosome injection*

(Days 26–32) Before exosome injection, all mice underwent a CPP test. Mice were divided into three groups: saline (n = 7), siRNA exosome (n = 7), ncRNA-RVG exosome and siRNA-RVG exosome (n = 7). On days 26, 28, 30 and 32, mice were injected intravenously with 200 μg exosomes loaded with or without siRNAs respectively, and the control group was injected intravenously with equivalent doses of saline.

#### *Reinstatement*

(Days 33–34) On day 33, before being placed in the white chamber for 30 min, all mice received morphine (10 mg/kg, i.p.). On day 34, all mice underwent a 30 min CPP test.

### Statistical analysis

Statistical analysis was performed using the t-test. Data are presented as the means ± SEMs of at least three independent experiments. Differences were considered statistically significant at P < 0.05.

## Additional Information

**How to cite this article**: Liu, Y. *et al*. Targeted exosome-mediated delivery of opioid receptor Mu siRNA for the treatment of morphine relapse. *Sci. Rep*. **5**, 17543; doi: 10.1038/srep17543 (2015).

## Supplementary Material

Supplementary Information
