# Supplementary material for: Targeted exosome-mediated delivery of opioid receptor Mu siRNA for the treatment of morphine relapse
Source: Sci Rep. 2015 Dec 3;5:17543. doi: 10.1038/srep17543 (PMC4668387; doi:10.1038/srep17543)
Supplement: Supplementary Information [file srep17543-s1.doc]

**Targeted exosome-mediated delivery of opioid receptor Mu siRNA for the treatment of morphine relapse**

**Yuchen Liu ^a, #^, Dameng Li ^a, #^, Zhengya Liu ^a, #^, Yu Zhou ^a^, Danping Chu ^a^, Xihan Li ^a^, Xiaohong Jiang ^a^, Dongxia Hou ^a^, Xi Chen ^a^, Yuda Chen ^a^, Zhanzhao Yang ^a^, Ling Jin ^a^, Waner Jiang ^a^, Chenfei Tian ^a^, Geyu Zhou ^a^, Ke Zen ^a^, Junfeng Zhang ^a, *^, Yujing Zhang ^a, *^, Jing Li ^a, *^ Chen-Yu Zhang ^a, *^**

^a^Jiangsu Engineering Research Center for microRNA Biology and Biotechnology, State Key Laboratory of Pharmaceutical Biotechnology, School of Life Sciences, Nanjing University, 22 Hankou Road, Nanjing, Jiangsu 210093, China

**Supplementary information**

**
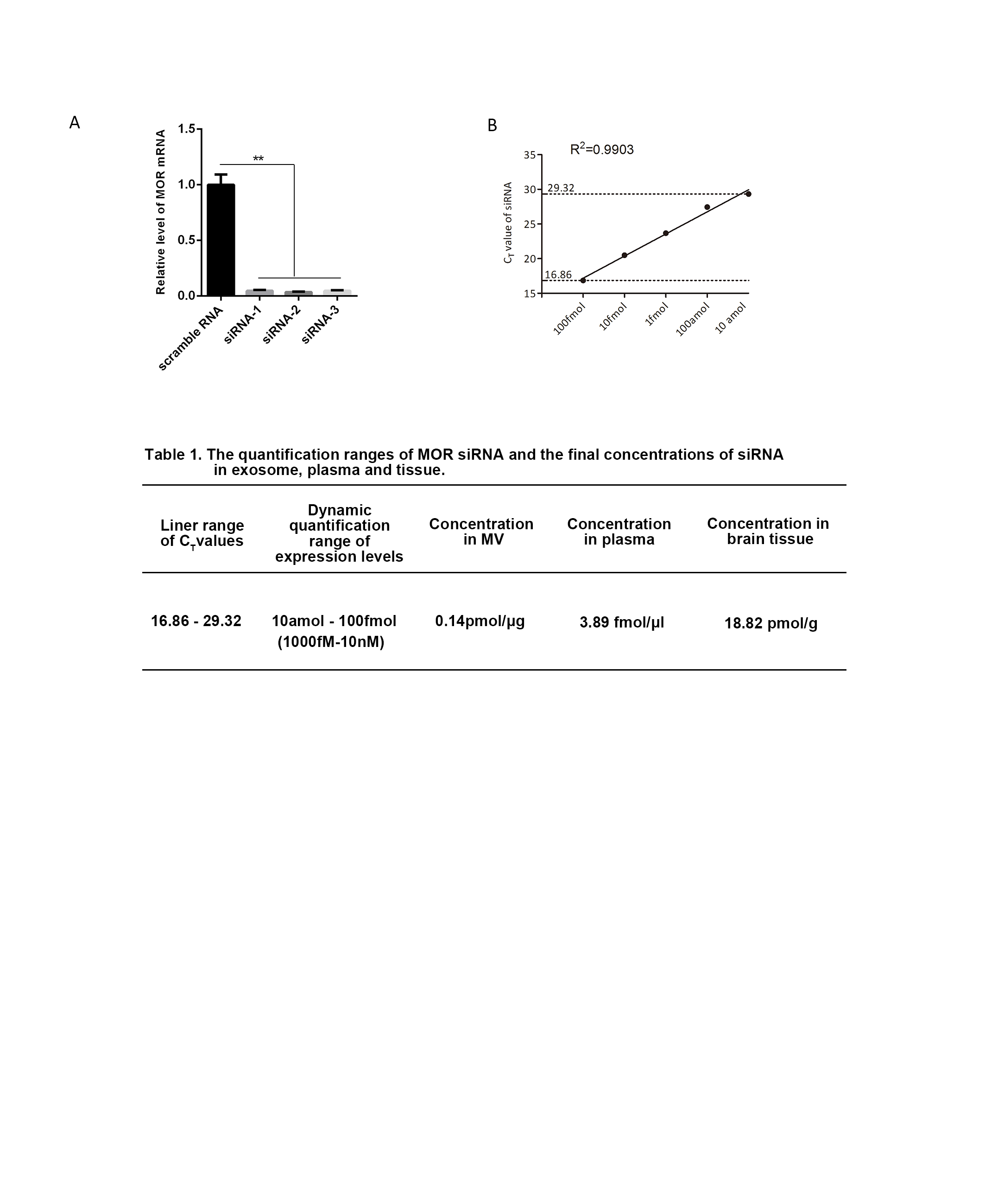
**

Supplementary figures 1

1. qRT-PCR analysis of MOR mRNA levels in 293T cells transfected with MOR siRNA.
2. qRT-PCR analysis of series diluted siRNA.


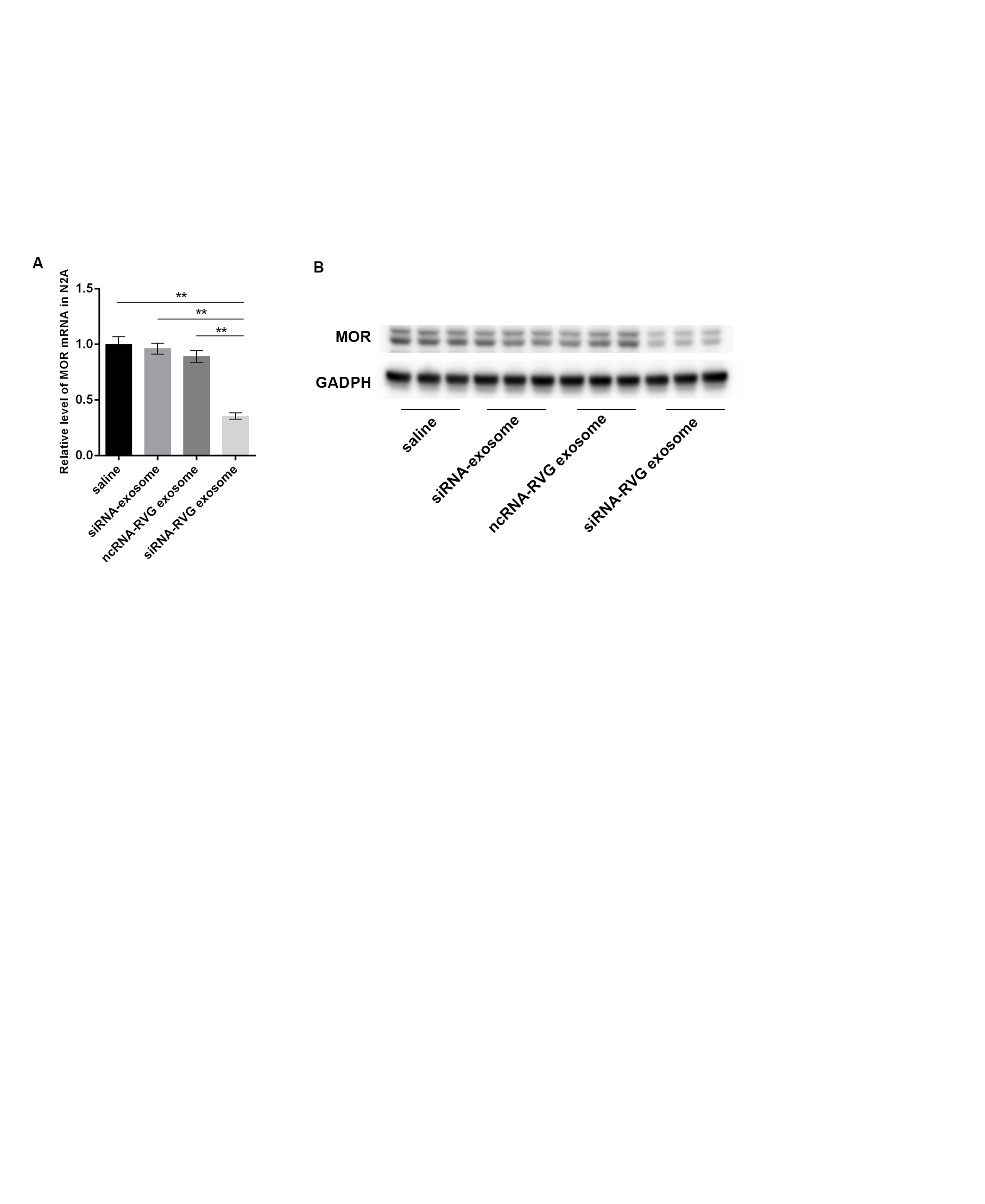


Supplementary figures 2

1. qRT-PCR analysis of MOR mRNA levels in N2A cells line untreated or treated with siRNA-2 in normal exosome, scramble RNAs in RVG exosome or siRNA-2 in RVG exosome.
2. Western blot analysis of MOR protein levels of Neuro2A cells treated as described in (A).


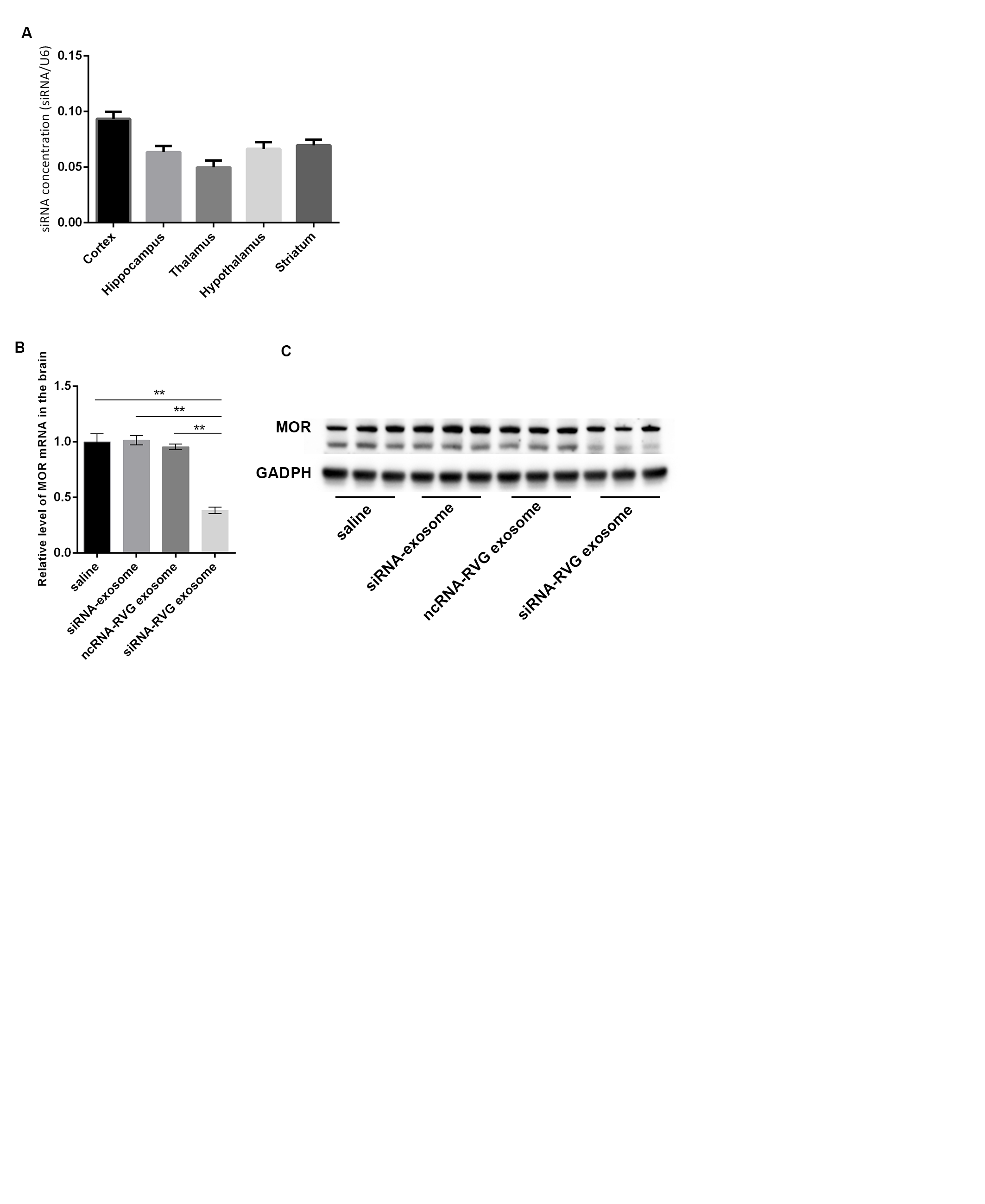


Supplementary figures 3

1. qRT-PCR analysis of siRNAs levels in Cortex, hippocampous, thalamus, hypothalamus, striatum. of the mice injected with siRNAs-loaded RVG exosomes.
2. qRT-PCR analysis of MOR mRNA levels in brain untreated or treated with siRNA-2 in normal exosome, scramble RNAs in RVG exosome or siRNA-2 in RVG exosome.
3. Western blot analysis of MOR protein levels of Neuro2A cells treated as described in (B).


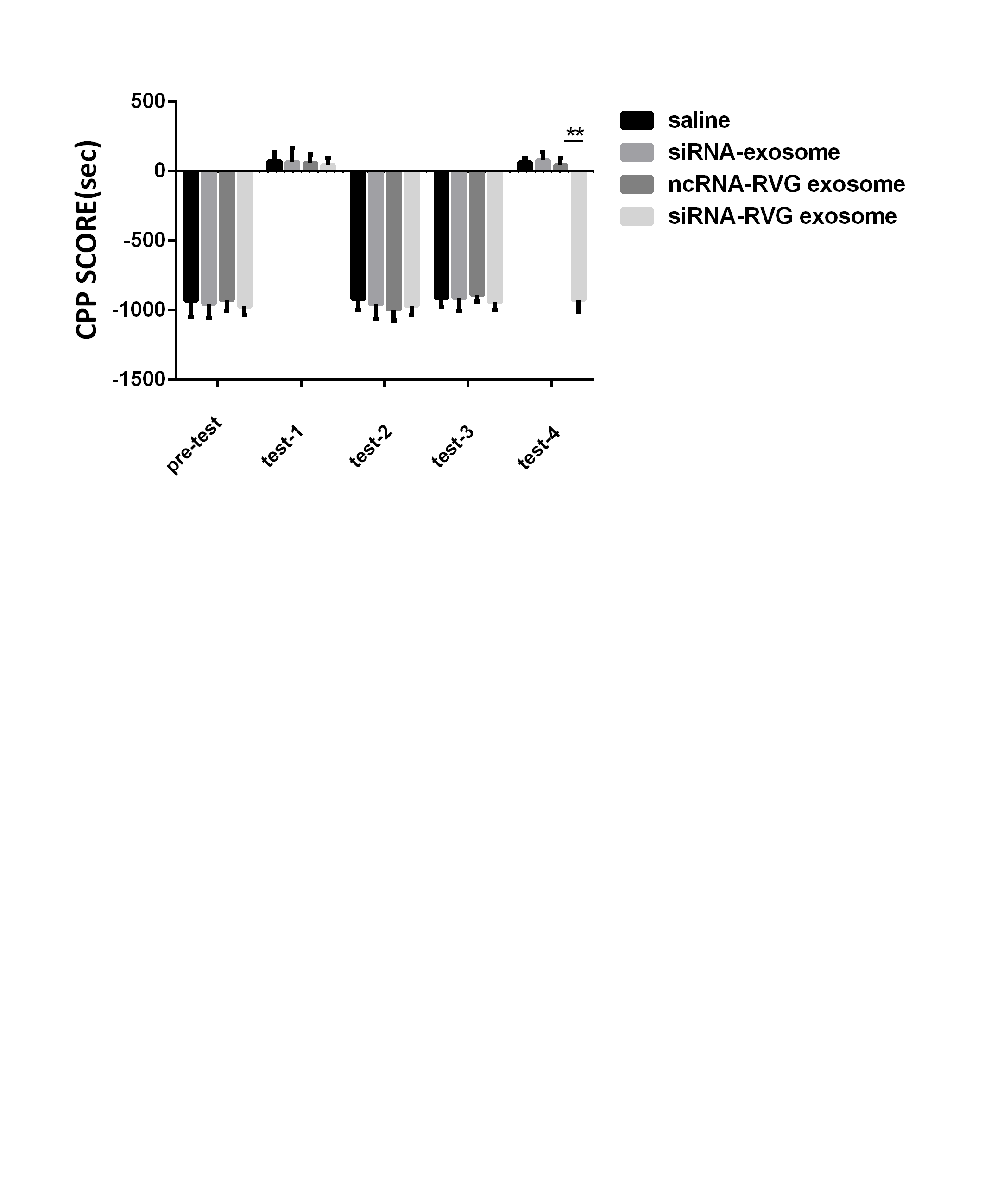


Supplementary figures 4

Analysis of the morphine-reduced CPP of mice treated with saline, empty exosomes, scramble RNAs in RVG exosomes or siRNAs RVG exosomes.
